# Wavelet analysis of circadian and ultradian behavioral rhythms

**DOI:** 10.1186/1740-3391-11-5

**Published:** 2013-07-01

**Authors:** Tanya L Leise

**Affiliations:** 1 Department of Mathematics, Amherst College, Amherst, MA 01002 USA

**Keywords:** Time series analysis, Fourier transform, Wavelet transform, Circadian rhythms, Ultradian rhythms, Rodent locomotor activity, Estrous cycle

## Abstract

We review time-frequency methods that can be useful in quantifying circadian and ultradian patterns in behavioral records. These records typically exhibit details that may not be captured through commonly used measures such as activity onset and so may require alternative approaches. For instance, activity may involve multiple bouts that vary in duration and magnitude within a day, or may exhibit day-to-day changes in period and in ultradian activity patterns. The discrete Fourier transform and other types of periodograms can estimate the period of a circadian rhythm, but we show that they can fail to correctly assess ultradian periods. In addition, such methods cannot detect changes in the period over time. Time-frequency methods that can localize frequency estimates in time are more appropriate for analysis of ultradian periods and of fluctuations in the period. The continuous wavelet transform offers a method for determining instantaneous frequency with good resolution in both time and frequency, capable of detecting changes in circadian period over the course of several days and in ultradian period within a given day. The discrete wavelet transform decomposes a time series into components associated with distinct frequency bands, thereby facilitating the removal of noise and trend or the isolation of a particular frequency band of interest. To demonstrate the wavelet-based analysis, we apply the transforms to a numerically-generated example and also to a variety of hamster behavioral records. When used appropriately, wavelet transforms can reveal patterns that are not easily extracted using other methods of analysis in common use, but they must be applied and interpreted with care.

## Introduction

Behavioral rhythms of animals span a wide range of cycle lengths, including circannual rhythms that vary with the seasons (period of 1 year), changes in activity due to the estrous cycle in rodents (cycle length of 4-5 days), circadian rhythms that track the daily light-dark cycle (period of 1 day), and ultradian rhythms of activity occurring within a single day (typically periods of 8 h or less).

The mammalian circadian pacemaker, the suprachiasmatic nucleus (SCN), governs circadian rhythms of tissues throughout the body as well as of outputs like activity, coordinating physiological processes internally and with the external environment by entraining to light-dark (LD) cycles [1]. Locomotor activity offers a convenient and non-intrusive way to measure the circadian rhythms of an animal, for example, by measuring wheel-running or by using a motion sensor. Behavioral rhythms can exhibit a circadian period (reflecting the circadian clock in the SCN) as well as ultradian periods. The ultradian rhythms emerge as a consequence of multiple physiological processes, not currently well understood, and tend to have greater interindividual variability than circadian rhythms [2]. In addition, the waveform of activity is known to vary under different conditions. For example, the waveform of animals entrained to an LD cycle often depends on the photoperiod, while hamsters under constant light (LL) can “split” their behavior and under 24 h LDLD cycles can “bifurcate” their activity rhythms [3]. These changes in waveform and the presence of ultradian rhythms point to the fact that behavioral records display a rich variety of patterns that we would like to be able to characterize and quantify.

The variability and noisiness of behavioral records creates a challenge in reliably determining period and phase of activity rhythms, and even more so in finding ways to quantify other aspects of behavioral patterns. In particular, behavioral records are typically nonstationary; their frequency content is not constant over time. A variety of methods have been applied to detect circadian rhythmicity and to measure the period of circadian rhythms for different types of molecular and behavioral data, including autocorrelation, Fourier and other periodograms, sine-fitting, cosinor analysis, maximum entropy spectral analysis (MESA), digital filtering, and wavelet-based methods [4-11]. Assessing characteristics of ultradian rhythms is particularly challenging, with few methods available. For instance, one study applied a continuous wavelet transform to identify how cage size affected ultradian rhythms in mice [12]. Similarly, a wavelet scalogram can be used to detect circadian and ultradian patterns in arterial pressure [13]. More recently, digital filtering, autocorrelation, and MESA have been used to analyze ultradian rhythms in the sleep-wake behavior of rats [14], and gender differences in circadian and ultradian behavioral rhythms have been explored with the use of cosinor analysis [15,16]. Other types of methods have also been applied to examine patterns in activity, including detrended fluctuation analysis to reveal a scale-invariance across the spectrum from seconds to 24 h [17].

In the following section, we briefly describe several methods of time-frequency analysis, specifically the Fourier periodogram and discrete and continuous wavelet transforms, and apply them to a numerically-generated time series with known circadian and ultradian periods to illustrate their use. In the Examples and discussion section, we apply the wavelet transforms to activity records from hamsters to demonstrate their efficacy on real data. We conclude with some final remarks, emphasizing a few caveats regarding the effective application of wavelet transforms.

## Fourier and wavelet time-frequency analysis methods

We expect that behavioral patterns will differ between the day and the night, at the very least in magnitude but also possibly in ultradian period. For instance, activity bouts may be briefer and occur more (or less) often during subjective day than during subjective night for a nocturnal rodent. How can we identify these sorts of patterns in an activity rhythm?

### Periodograms

The natural place to start when carrying out a mathematical analysis of frequency is a Fourier periodogram. For a record with many cycles, a periodogram can yield good estimates of the dominant frequencies occurring in a stationary time series.

**Figure 1:**
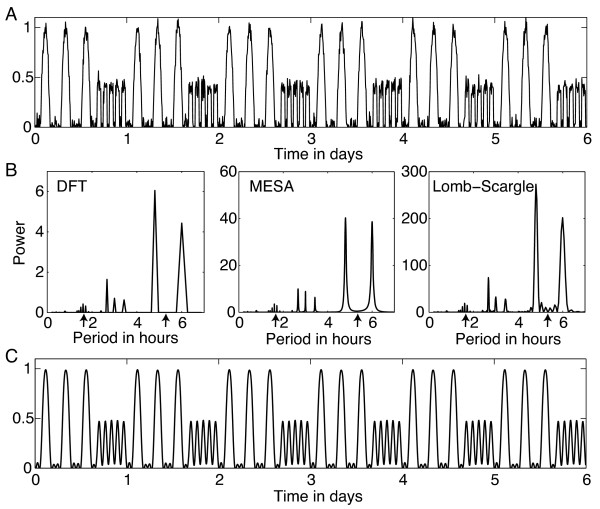
**Periodogram analysis of a time series. (A)** Simulated time series with 16 hours of period 5.3 h alternating with 8 hours of period 1.62 h, plus white noise. **(B)** Fourier, MESA, and Lomb-Scargle periodograms all have similar large spikes at harmonics 24/4=6 h and 24/5=4.8 h, plus small spikes at harmonics 24/7=3.43 h, 24/8=3 h, and 24/9=2.67 h. Note the absence in all 3 periodograms of significant power at the ultradian periods 5.3 h and 1.62 h, marked by small arrows along the horizontal axis. **(C)** Inverse DFT of the first 18 harmonics. While the periodograms do not provide a direct means of detecting the ultradian periods of interest, if the underlying ultradian pattern is sufficiently regular (as is the case in this example), then taking the inverse DFT of the circadian harmonics can reveal what that pattern is.

Let a time series be generated by sampling a process every *Δt* hours, with *x*_*k*_ the measurement taken after *kΔt* hours have elapsed (e.g., if activity is binned every 6 minutes, then *Δt* = 0.1 h). If *N* such samples are taken, this uniform sampling results in the finite sequence $\left\{x_{0},x_{1},\ldots ,x_{N-1}\right\}$. The discrete Fourier transform (DFT) $\left\{{\hat{x}}_{0},{\hat{x}}_{1},\ldots ,{\hat{x}}_{N-1}\right\}$ of this time series is defined by

(1) $${\hat{x}}_{k}=\overset{N-1}{\underset{n=0}{\sum }}x_{n}e^{-2\mathrm{\pi ikn}/N},$$

where $i=\sqrt{-1}$. The Fourier periodogram shown in Figure 1 for a numerically-generated time series displays the power spectral density ${\hat{x}}_{k}^{2}/N$ corresponding to period *NΔt*/*k* hours.

To understand what ultradian frequencies the DFT is able to detect, let’s examine Equation (1) in the context of a circadian rhythm. Suppose the time series has a period of *τ* hours (with possibly some ultradian periods as well), so *x*_*n*_=*x*_*n* mod *s*_ for all *n*, where there are *s* = *τ*/*Δt* time points per day. Also assume that the times series covers *D* periods, so *N* = *Ds*. Then we can break up the DFT sum into portions covering each of the *D* days, on each of which the time series $\left\{x_{0},\ldots ,x_{s-1}\right\}$ repeats itself:

$$\begin{array}{l} {\hat{x}}_{k}=\overset{D-1}{\underset{d=0}{\sum }}\overset{s-1}{\underset{n=0}{\sum }}x_{n}e^{-2\mathrm{\pi ik}(n+\text{ds})/N}\;\\ \;=\overset{s-1}{\underset{n=0}{\sum }}x_{n}e^{-2\mathrm{\pi ikn}/N}\overset{D-1}{\underset{d=0}{\sum }}e^{-2\mathrm{\pi ikd}/D}.\; \end{array}$$

We can apply the geometric sum formula, $\sum_{d=0}^{D-1}r^{d}=\frac{1-r^{D}}{1-r}$ for *r*≠1, with *r* = *e*^−2*πik*/*D*^, leading to

$$\begin{array}{lll} {\hat{x}}_{k} & =\overset{s-1}{\underset{n=0}{\sum }}x_{n}e^{-2\mathrm{\pi ikn}/N}\cdot \frac{1-e^{-2\mathrm{\pi ikD}/D}}{1-e^{-2\mathrm{\pi ik}/D}}\; & \;\\ & =\overset{s-1}{\underset{n=0}{\sum }}x_{n}e^{-2\mathrm{\pi ikn}/N}\cdot \frac{1-1}{1-e^{-2\mathrm{\pi ik}/D}}\; & \;\\ & =0\; & \; \end{array}$$

if *k* is not a multiple of *D* (noting *e*^−2*πim*^=1 for all integers *m*). Therefore the DFT coefficients ${\hat{x}}_{k}$ are only nonzero when *k* = *mD* for some positive integer *m*, which correspond to periods $\frac{\mathrm{N\Delta t}}{k}=\frac{\tau }{m}$ hours. That is, only harmonics of *τ* (the period of the daily rhythm) can appear in the DFT, and so the true ultradian periods will not be revealed by the periodogram. This fact is reflected in Figure 1B, which shows DFT spikes at harmonics of 24 h, but no spikes at the actual ultradian period values. Also note that a square wave with period 24 h will have spikes at all harmonics of 24 h (12 h, 8 h, 6 h, etc), even though that signal involves no ultradian periods. Therefore the presence of spikes in the DFT at harmonics does not directly indicate whether or not ultradian periods are present.

Note that the periodogram applied to real data will display some frequencies other than the harmonics of *τ* because real activity records are noisy and vary from day to day, so they don’t perfectly repeat a pattern every cycle. However, if the interest lies in extracting patterns that do essentially repeat daily, this analysis implies that the Fourier periodogram will not be useful in measuring the true period(s) of ultradian activity patterns (even if an ultradian period coincides with a harmonic, we have no way of easily distinguishing whether or not a large spike at a harmonic indicates a true ultradian period). Other periodograms present a similar difficulty for measuring ultradian periods, as illustrated in Figure 1B. In general, methods like the DFT are not well-suited for nonstationary time series.

The DFT has an advantage over other periodogram methods in that it can be inverted. If the circadian pattern of activity is sufficiently regular, like in the simulated time series in Figure 1, then we can keep the dominant harmonics (with periods *τ*/*m*) from the DFT and invert to see what this pattern is, as shown in Figure 1C. In practice, this approach works best for animals with very predictable timing of activity bouts; the discrete wavelet transform described below offers a more flexible tool for this purpose.

It is important to keep in mind that the purpose of periodograms like those shown in Figure 1B is to determine frequencies present *globally* in the signal, so they do not provide the proper tool for the problem of determining ultradian frequencies present during particular time intervals, particularly if the period can differ during, say, subjective day and night for an animal, or for detecting changes in the circadian period from day to day. A method that can localize in time is more appropriate for these tasks, which involve nonstationary time series. The classic example of such time-frequency analysis is the wavelet transform, which comes in two flavors, discrete and continuous. The continuous wavelet transform provides a replacement for the periodograms, by offering high resolution period information that is localized in time. The discrete wavelet transform provides an alternative method to inverting the DFT for identifying the daily pattern of activity bouts, with the flexibility that it does not require bouts be similarly timed each day.

We should note that, while wavelet transforms can provide excellent resolution of how the frequency or period changes over time, all time-frequency analysis must obey the limitations imposed by the Heisenberg uncertainty principle, which in essence says that increasing the time resolution will decrease the frequency resolution, and vice versa. Just as we cannot simultaneously know the exact position and momentum of a quantum particle, in the signal processing context we cannot simultaneously pinpoint time and frequency. The choice of wavelet determines how sensitive the corresponding wavelet transform can be to frequency as opposed to time specificity, but there is no way to obtain perfect resolution in both time and frequency.

### The continuous wavelet transform

Continuous wavelet transforms convolve a time series *x*(*t*) with a wavelet function *ψ*(*t*), essentially finding the correlation between the time series at different points in time with scaled versions of the wavelet function to determine the frequency that best describes the times series at each point in time. See [18] for an introduction to wavelet analysis. Continuous wavelet transforms can use real-valued wavelet functions, like the Mexican Hat wavelet used in [11] to analyze body temperature rhythms, or they can be complex-valued, like the Morlet wavelet used in [13] to analyze bioluminescence rhythms for molecular data (e.g., PER2::LUC oscillations). Complex-valued wavelet transforms yield both amplitude and phase information over time, while real-valued wavelets can be better at isolating peaks and discontinuities [18]. Choice of which wavelet function to use also depends on the desired resolution in time versus frequency, and on the characteristics of the time series, e.g., smooth and sinusoidal or choppy and discontinuous. A wavelet function whose shape reflects the features of the data often works best. Experimentation with different choices can indicate which wavelet is best suited for a particular set of data.

Here we focus on a complex-valued wavelet function that is analytic (meaning the Fourier transform equals zero for negative frequencies) called the Morse wavelet function [19], so the resulting wavelet transform

(2) $$W_{\psi }(t,s)=\int_{-\infty }^{\infty }\frac{1}{s}\psi^{*}\left(\frac{u-t}{s}\right)x(u)\text{du}$$

is referred to as an *analytic wavelet transform* (AWT). (The asterisk indicates the complex conjugate, and we have used bandpass normalization to define the transform.) The time *t* refers to the current time point of interest in the time series *x*(*t*), and the scale *s* corresponds to the period 2*πs*/*ω*_*ψ*_, where *ω*_*ψ*_ is the mean frequency of the Morse wavelet function *ψ*(*t*). The heat map of the magnitude |*W*_*ψ*_(*t*,*s*)| yields information about the frequencies present in the time series at each point in time and the amplitude associated with those frequencies. The *wavelet ridges* run along the local maxima *s* = *s*_*max*_(*t*) of |*W*_*ψ*_(*t*,*s*)|, indicating the scale *s*_*max*_(*t*) that yields the greatest correlation with the time series at time *t* and thereby estimates the instantaneous period 2*πs*_*max*_(*t*)/*ω*_*ψ*_. The value of |*W*_*ψ*_(*t*,*s*_*max*_(*t*))| equals the amplitude of the rhythm at time *t* associated with the instantaneous period, while the complex argument (polar angle) of *W*_*ψ*_(*t*,*s*_*max*_(*t*)) indicates the phase at time *t*. Consistent with normal usage in mathematics, we define the *amplitude* of an oscillation to equal the distance between the midpoint value and the maximum value. For example, *A* is the amplitude of the sinusoidal function *A* cos(*ωt*)+*C*. Doubling the amplitude gives the peak-to-trough height of the oscillation.

**Figure 2:**
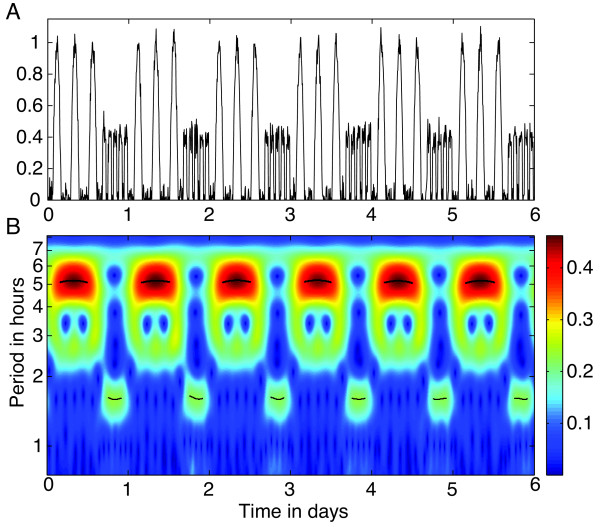
**AWT of the simulated activity time series.** The heat map in **(B)** indicates the absolute value of the AWT coefficients for the simulated time series, shown again in **(A)** for ease of comparison. The short black curves in **(B)** are the wavelet ridges that provide an estimate of the instantaneous period, averaging 5.2 h and 1.6 h during the alternating intervals (close to the true values 5.3 h and 1.62 h). The amplitude is indicated by the color of the heat map. The amplitude of the time series alternates between 0.5 and 0.2, so the AWT also correctly estimates the amplitude during each time interval.

As an illustrative example, examine the AWT in Figure 2 of the simulated time series from Figure 1A. For a finite length, discrete time series $\left\{x_{0},x_{1},\ldots ,x_{N-1}\right\}$, we use a discretized version of (2), as explained in [18]. Observe that the period in the AWT heat map is not scaled linearly along the vertical axis, since the transform is calculated with respect to scale *s*, the reciprocal of period, but here we have converted to period for ease of interpretation. For this example, the AWT closely estimates the periods 5.3 h and 1.6 h of the alternating ultradian rhythms, and also correctly estimates their amplitude. The AWT can tell us both *what* periods are present in the times series and *when* they occur (within the constraints of the uncertainty principle).

The AWT must be interpreted with care. If the activity of an animal is too variable, the AWT may not yield anything usable. It suffers problems with harmonics, which appear as “echoes” in the heat map below the hot spots marking dominant frequencies. Wavelet transforms, like other filtering techniques applied to finite length time series, exhibit edge effects due to the wrap-around nature of the filtering process. Edge effects can be minimized for activity data by starting and ending the time series to be transformed at midpoints of rest intervals. See [9,18] for further discussion of edge effects.

### The discrete wavelet transform

The discrete wavelet transform (DWT) is rather different in nature from the continuous version. In place of a wavelet function, a high-pass wavelet filter and a low-pass scaling filter are repeatedly applied to yield a set of *wavelet details*$D_{1},\ldots ,D_{J}$ (as well as wavelet smooths, which we won’t discuss). The sum of the wavelet details plus the final smooth equals the original time series, so the DWT decomposes the time series into components associated with certain period ranges. More specifically, each wavelet detail *D*_*j*_ is associated with a frequency band corresponding to periods approximately 2^*j*^*Δt* through 2^*j*+1^*Δt* (as before, we sample every *Δt* hours to generate the time series), assuming we use certain families of filters like the Daubechies filters. For instance, if the time series is an activity record with 6 minutes bins (*Δt* = 0.1 h), then *D*_5_ covers roughly the period range 3.2-6.4 h. The value of *Δt* controls the range of periods associated with each wavelet detail, so we can adjust that range through the bin size. If the circadian component is desired, then choosing *Δt* = 0.25 h (15 minute bins) works well so that *D*_6_ corresponds to the period range 16-32 h. If a particular ultradian rhythm is sought, then it can be helpful to choose a bin size so that the period range of one of the details is centered on the desired period.

For this application, we chose a translation-invariant DWT with the Daubechies least asymmetric filter of length 12, sometimes called *symlet6*. Shorter length filters result in more overlap between the frequency bands associated with each wavelet detail, so that the components are not as well separated with respect to the period ranges. Longer length filters can worsen edge effects. See [20] for an in-depth explanation of the translation-invariant DWT (also known as a maximal overlap DWT), and see [9,21] for practical overviews.

**Figure 3:**
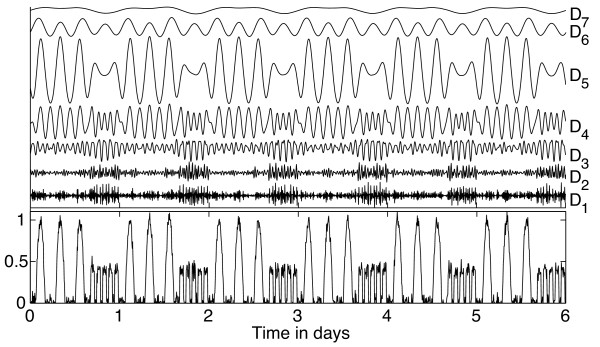
**DWT of the simulated activity time series shown in Figure **1A, using the ***symlet6 *filter.** The wavelet details *D*_1_ through *D*_7_ are shown at the same scale as the time series itself so that the magnitudes can be directly compared.

**Figure 4:**
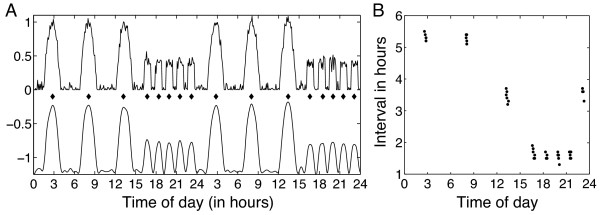
**Bout interval analysis using the DWT. (A)** The first 2 days of the original simulated time series from Figure 1A is shown above the cleaned-up version, which equals the sum of the wavelet details D_3_-D_7_ from Figure 3. Peaks of the summed wavelet details (marked by diamonds) correspond to midpoints of the simulated activity bouts. **(B)** Length of time intervals between midpoints of activity bouts, measured as the distance between peaks of the summed wavelet details. The three large bouts of activity are separated by roughly 5.3 h (perturbed by the added noise), the four shorter bouts of activity are separated by around 1.6 h, and the transition intervals are around 3.5h. These values are correctly identified by the DWT interval analysis.

Again consider the simulated time series in Figure 1A to illustrate possible uses of the DWT for analysis of activity records. Figure 3 shows the wavelet details obtained from the DWT of the simulated time series, for which *Δt* = 0.1 h. Wavelet detail *D*_5_ (period range 3.2-6.4 h) reflects the large activity bouts with ultradian period 5.3 h, while *D*_3_−*D*_4_ (period ranges 0.8-1.6 h and 1.6-3.2 h, respectively) best reflect the ultradian rhythm with period 1.6 h. To capture the overall pattern occurring in the time series, we sum *D*_3_−*D*_7_ together (roughly covering period range 1-26 h), shown underneath the time series in Figure 4A. This offers a more flexible version of the DFT method shown in Figure 1C, as DWT approach continues to yield good results even if the timing of the bouts varies from day to day. To measure the ultradian periods present during each part of the day, we can examine the time intervals between peaks of the summed wavelet details (which roughly correspond to midpoints of activity bouts). These intervals are plotted in Figure 4B, demonstrating that this approach can capture the ultradian periods present during different parts of the day.

The DWT is also effective at detecting sharp discontinuities in a time series (with an appropriate choice of filter), such as occur with activity onsets. See [11] for a description and examples of how the DWT can be used to detect onsets in activity records, which is not discussed in this review.

### Computations

The freely available MATLAB wavelet toolbox *jlab*[22] was used to compute the AWT (using *β* = 3 and *γ* = 8 in the Morse wavelet function), and the freely available MATLAB wavelet toolbox *wmtsa*[23] (companion software for [20]) was used to compute the translation-invariant DWT (which refers to *symlet6* as *la12*). All calculations for both the simulated time series in the Methods section and for the real data sets described in the Results section were run in MATLAB 8.0.0.783 (The MathWorks, Natick, MA).

### Animal care

Regarding the activity records of Syrian hamsters from Eric Bittman’s lab: All procedures were approved by the animal care and use committee (IACUC) of the University of Massachusetts at Amherst, and conform to all USA federal animal welfare requirements.

Regarding the activity records of Syrian hamsters from Brian Prendergast’s lab: All procedures conformed to the USDA Guidelines for the Care and Use of Laboratory Animals and were approved by the Institutional Animal Care and Use Committee (IACUC) of the University of Chicago.

## Examples and discussion

To demonstrate that the AWT and DWT can be effective in analyzing real behavioral data, we apply the methods described in the previous section to a variety of hamster activity records. We also discuss some of the difficulties that can be encountered when applying these transforms for real data.

### Tracking changes in activity over the estrous cycle

**Figure 5:**
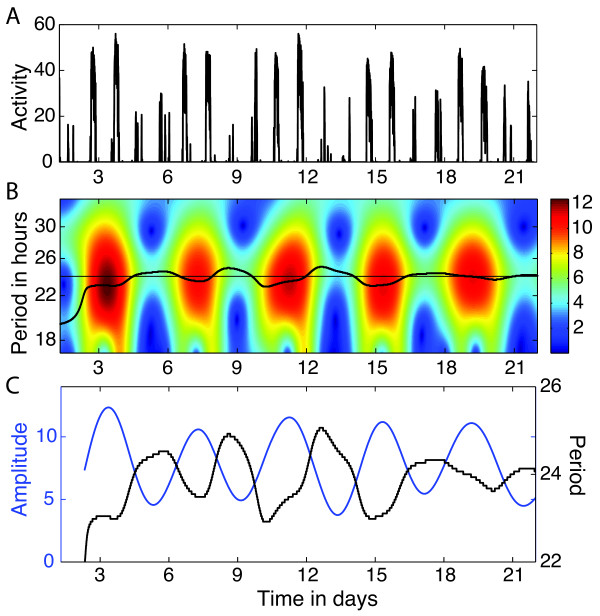
**Example of the AWT applied to detect changes in period and amplitude over time. (A)** Time series of wheel running (counts per 6 minute bin) for a female hamster in constant darkness. **(B)** Heat map of the magnitude of the AWT coefficients. The black curve is the wavelet ridge that indicates the instantaneous period, while the color of the heat map indicates amplitude. **(C)** Curves showing the amplitude (in blue) and period (in black), extracted from the wavelet ridge in **(B)**, revealing that the oscillation of the amplitude is nearly antiphase to the oscillation of the period in this example. Hamster record courtesy of Eric Bittman and Emily Manoogian.

The estrous cycle in hamsters typically results in an approximately 4-day pattern in the amplitude and period of activity (“scalloping”), due in part to the effects of estradiol [24]. The AWT can be effective in tracking these changes in amplitude and period over time, if the record is sufficiently long. The difficulty is that edge effects can distort the AWT heat map, so that 1-2 days at the beginning and end are not reliable. If a 4-day pattern is being sought, then the activity record should cover at least 2 uninterrupted weeks, preferably more, for the AWT to yield good results. A further disadvantage of the AWT is that missing data in the record can also distort the results. Nevertheless, on uninterrupted records of sufficient length, the AWT can provide a spectacular visualization of the effects of the estrous cycle on activity. See Figure 5 for an example. For other examples of using wavelet analysis to detect period and amplitude changes across the estrous cycle, see [9] (in mice) and [11] (in hamsters).

### Ultradian periods during day and night

**Figure 6:**
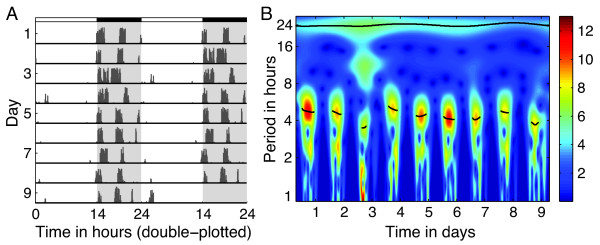
**Example of the AWT applied to detect an ultradian period of activity. (A)** Actogram displaying wheel running (6 minute bins) for a male hamster entrained to 14L:10D. **(B)** Heat map of the magnitude of the AWT coefficients. The wavelet ridge (the black curve) indicates the instantaneous period, while the color of the heat map indicates amplitude. While in the light, the hamster is not active so the AWT heat map shows dark blue, while in darkness the hamster typically displays an ultradian period around 4.5-5 h (indicated by the wavelet ridges in black on the hot spots). On day 3, two large bouts of activity are closely spaced, resulting in a lower ultradian period and a strong 12 h harmonic. The wavelet ridge at period 24 h indicates that the animal is successfully entrained to the 24 h LD cycle. The yellow spots around period 2 h are for the most part harmonics of the hotspots above them. Hamster record courtesy of Eric Bittman and Emily Manoogian.

The wavelet-based analysis can also be effective at detecting changes in ultradian period across the day. For example, we can apply the AWT to a hamster wheel-running record to detect a roughly 5 h ultradian period during the night, as shown in Figure 6. Observe that the AWT heat map only shows hot spots during the night and not during the day, correctly reflecting that the activity is only occurring when the lights are off. Note that the ridge curves will not yield good estimates of the times of activity onset and offset, as the border between time intervals of different frequencies (e.g., activity patterns during subjective day versus subjective night) will appear smeared, due to the limitations of time versus frequency resolution. For wavelet-based determination of the precise time of activity onset, use a method like that described in [11].

**Figure 7:**
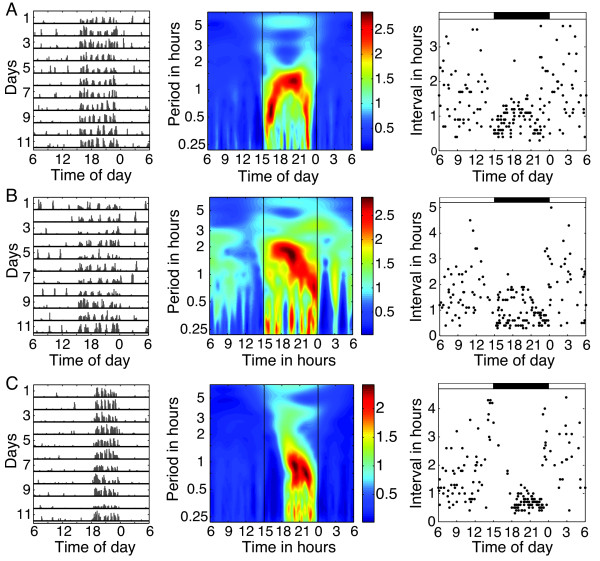
**Actograms displaying motion (6 minute bins) from 3 male hamsters entrained to 15L:9D, with averaged AWT heat maps and graphs of DWT-derived intervals between midpoints of activity.** The AWT heat maps show the mean of the absolute value of the AWT coefficients, taken across 11 days. **(A)** The activity of this hamster exhibits a very short period near lights on and off and a longer ultradian period during the middle of the night. This pattern is reflected in both the AWT and the DWT-derived interval analysis. During the day, the bouts appear more randomly spaced, with no clear frequency emerging in either the AWT heat map or the interval graph, a pattern which also appears in the other two records. **(B)** This hamster appears to display two ultradian frequencies at night, a very short period of less than 0.5 h and a longer period that starts around 2 h shortly after lights-off and decreases through the night, again reflected in both the AWT heat map and the DWT-derived intervals analysis. **(C)** This hamster shows almost no activity in the first part of the night, after which it exhibits ultradian periods around 1 h and less than 0.5 h. Hamster records courtesy of Brian Prendergast.

As another example, consider the three hamster records shown in Figure 7. We apply both the AWT and DWT methods to these records. To minimize the obscuring effects of day-to-day variations, the AWT heat maps are averaged over the 11 days of the record. Comparison of the two methods is recommended, as agreement between them increases confidence that the results are meaningful. During the night when activity is much greater, the ultradian patterns revealed by the wavelet-based techniques are clear, though with some random variation in the actual period. On the other hand, activity is sparse and sporadic when light is present, with the intervals varying from 0.5-3 h. Whether the lack of a clear ultradian period during daytime is due to masking suppressing the animal’s activity, is because the animal doesn’t express a coherent ultradian rhythm during its rest phase, or is caused by some other factor requires further investigation.

## Final remarks

The AWT and DWT offer alternatives to try when other techniques prove insufficient to analyze a time series in the desired manner. We don’t suggest that wavelet transforms be the first techniques to apply when studying a new set of behavioral records, as well-established methods are in many cases sufficient to answer the questions of interest. Wavelet-based methods must be applied and interpreted with care, keeping in mind issues with harmonics and edge effects. In particular, the record must be sufficiently long so that a day or so can be discarded on each end of the resulting wavelet transform since these portions may be distorted by edge effects. If a time series is excessively noisy, has too much missing data, or the rhythms are not focused on particular frequencies, the wavelet transforms may not yield anything useful. However, when used appropriately on relevant datasets, the AWT and DWT can reveal patterns that are not easily extracted using other methods of analysis in common use, thereby expanding the types of questions we can ask a set of behavioral records to answer. The methods presented here offer a means of identifying circadian and ultradian patterns and how they change over time, from day-to-day as well as over the course of a day.

## Abbreviations

AWT: Analytic wavelet transform; DFT: Discrete fourier transform; DWT: Discrete wavelet transform; LD: Light-dark; MESA: Maximum entropy spectral analysis.

## Competing interests

The author declares that she has no competing interests.
